# Family Functioning Styles and Exercise Addiction: Disengaged, Enmeshed, and Rigid Family Patterns Are Associated with Exercise Addiction

**DOI:** 10.3390/ejihpe14010010

**Published:** 2024-01-03

**Authors:** Alessio Gori, Eleonora Topino, Mark D. Griffiths

**Affiliations:** 1 Department of Health Sciences, University of Florence, Via di San Salvi 12, Pad. 26, 50135 Florence, Italy; 2 Department of Human Sciences, LUMSA University of Rome, Via della Traspontina 21, 00193 Rome, Italy; e.topino@lumsa.it; 3 Psychology Department, Nottingham Trent University, 50 Shakespeare Street, Nottingham NG1 4FQ, UK; mark.griffiths@ntu.ac.uk

**Keywords:** exercise, exercise addiction, family functioning, body image concerns, behavioural addiction

## Abstract

Physical exercise is a widely recommended practice for promoting health, but for some individuals, this activity can result in pathological and morbid behaviour. Therefore, the study of the factors contributing to the onset, development, and progression of exercise addiction is particularly relevant. Within this framework, the present study assessed the effect of family functioning, body image concerns, age, and gender on exercise addiction. A sample of 300 regular exercisers (*M_age_* = 30.3 years, *SD* = 11.6; 69.7% females, 30.3% males) participated in the study and completed the Family Adaptability and Cohesion Evaluation Scales–IV, Body Image Concern Inventory, and Exercise Addiction Inventory. Data were analysed by implementing a series of moderated moderated-mediations. Results showed that three significant models were relevant. First, positive associations of disengaged (*p* < 0.05), enmeshed (*p* < 0.05), and rigid (*p* < 0.01) family functioning with exercise addiction were found. Furthermore, body image concerns mediated all these relationships, and the interaction between gender and age significantly moderated the effects of body image concerns on exercise addiction (*p* < 0.05). Such data may be useful for a deeper understanding of the variables associated with the development of exercise addiction, suggesting key elements on which it might be useful to focus in clinical and/or preventive activity.

## 1. Introduction

Regular exercise is widely considered to be beneficial for mental and physical well-being. Exercise promotes numerous benefits, including a lower risk of cardiovascular disease and mortality [1,2], improvements in mental health [3], and enhanced cognitive functioning and well-being [4]. However, if practiced in a dysregulated manner, exercise can become harmful [5,6] and manifest itself as a behavioural addiction [6,7]. Addicted exercisers exhibit an inability to control their exercise behaviours [7,8], which become compulsive and persistent, regardless of physical injury, personal discomfort, or problems in other areas of life (see Landolfi [9] for a review). It is characterized by the inability to abstain from exercise, and withdrawal symptoms occur when the activity cannot be performed. Therefore, while regular exercisers engage in healthy behaviours that improve psychophysical health [10,11], individuals with exercise addiction engage in dysregulated physical activity that results in a negative impact on both mental and physical functioning [12,13]. For example, exercise addiction has been associated with an increased risk of injury [14], reduced quality of sleep [15], as well as heightened levels of anxiety [16] and depression [17]. 

Although not included in the chapter on “Substance-Related and Addictive Disorders“ of the latest (fifth) edition of the *Diagnostic and Statistical Manual of Mental Disorders* (DSM-5 and DSM-5-TR) [18,19], several authors have engaged in the definition of exercise addiction features and the study of critical issues and associated effects. Among these, Griffiths [7] posited that all behavioural addictions comprise six core components, which are also applicable to exercise addiction: salience (i.e., the exercise becomes the most important activity in the individual’s life), mood modification (i.e., the subjective mood-modifying experience after engaging in exercise), tolerance (i.e., the need to increase the amount of exercise to achieve the initial mood-modifying effects), withdrawal symptoms (i.e., the unpleasant effects that occur when exercise is stopped or suddenly reduced), conflict (i.e., the interpersonal and intrapsychic conflicts with other activities or individuals due to exercise), and relapse (i.e., the tendency to repeat earlier patterns of maladaptive exercise, even after months or years of abstinence or control). 

Furthermore, an important element of distinguishing between healthy and pathological physical activity concerns the inability to stop exercising despite negative consequences at psychological, physiological, and social levels which interfere with the normal functioning of the individual [20,21]. Therefore, the study of the factors that contribute to its onset, development, and progression is particularly relevant [22]. Within this framework, the present study aimed to contribute knowledge to the field by exploring the association between risk factors for exercise addiction, specifically examining family functioning, body image concerns, age, and gender.

Previous evidence has highlighted the role of family functioning in being a risk factor or protective factor in the development of addiction [23]. Family functioning refers to the overall quality of family life [24], and its poor quality has been associated with a wide range of psychopathological problems [25,26] and a greater likelihood of engaging in risky behaviours [27], as well as early-onset of substance use and progression to heavy/problematic use [28]. Although previous research has highlighted significant associations between family functioning and some behavioural addictions such as smartphone addiction [29], problematic internet use [30], internet gaming disorder [31], and gambling disorder [32], the evidence of its influence on exercise addiction is still scarce. Among various models for conceptualizing family functioning, the Circumplex Model by Olson and colleagues [23,24] categorizes family patterns as balanced (characterized by functional levels of cohesion and flexibility) or unbalanced (which may manifest with high levels of disengagement, enmeshment, rigidity, and chaos). This perspective [24,33] has recently been used in the context of behavioural addictions [34]. Therefore, this model could be a useful conceptualization for exploring the associations between different patterns of family functioning and exercise addiction.

Body image perception refers to an individual’s subjective evaluation of their body as positive or negative [35], and it is partly influenced by parental input [36]. Previous research has shown that family functioning has an impact on different components of self-concept, including physical self-concept [37], and therefore represents a key element in the development of bodily satisfaction or dissatisfaction [38]. Poor body image is predictive of low self-esteem [39], depression [40], and development of eating disorders [41]. Furthermore, body image is a relevant factor in initiating, maintaining, and stopping physical activity [42], and body image concerns have been found to be positively associated with exercise addiction [43]. 

However, individuals’ dissatisfaction with their body image can manifest itself differently between males, mainly focused on muscle mass, and females, who instead may experience more of a drive toward thinness [44], and this could therefore influence exercise addiction. In this regard, among the studies examining the direct effect of gender in problematic exercise, some showed significant differences (sometimes with higher levels among females or, for the most part, with higher scores among males), while others have found no gender differences at all (see Dumitru, Dumitru, and Maher [45] for a review). The presence of conflicting results suggests the need to study the phenomenon more in-depth to shed insight into the role of gender in relation to exercise addiction. 

Age can also influence the effects of body satisfaction in light of the variations on the importance given to aspects related to the shape of the body, which in some studies appears to decrease as individuals become older [46] while in others it maintains its relevance even among individuals aged over 65 years [47]. Also, in this case, the evidence associated with exercise addiction does not follow a single direction because research examining the relationship with age has sometimes identified a greater risk among young people [48], in other cases among older individuals [49], and sometimes reported no effect at all [15]. The inconsistency found in the studies that consider gender and age suggests that these may not have a linear relationship regarding problematic exercise and it is therefore plausible to consider their mutual influence in the interaction with body image concerns.

Taking these aforementioned findings into account, the present study examined the relationship between family functioning and exercise addiction among regular exercisers, by considering the effect of body image concerns and their interaction with age and gender. To accomplish this, a series of moderated moderated-mediations were implemented to investigate the (i) relationship between the several patterns of family functioning and exercise addiction, (ii) mediating role of body image concerns in the associations between the several patterns of family functioning and exercise addiction, and (iii) moderated effect of the interaction between age and gender in the relationship between body image concerns and exercise addiction. 

It was hypothesized that adaptive family functioning styles (i.e., cohesion and flexibility) would have a negative association with exercise addiction, and that less adaptive functioning styles (i.e., enmeshed, disengaged, chaotic, and rigid) would have a positive association with exercise addiction. Furthermore, it was hypothesized that adaptive family functioning styles would have a negative association with body image concerns, and that less adaptive family functioning styles would have a positive association with body image concerns. Finally, it was hypothesized that body image concerns would be positively associated with exercise addiction, but it would be moderated by the interaction between gender and age.

## 2. Materials and Methods

### 2.1. Participants, Procedure, and Ethics

The sample comprised 300 Italian individuals who declared that they engaged in regular physical exercise. Their age ranged from 18 to 75 years (*M_age_* = 30.3 years, *SD* = 11.6) and most of them were female (69.7%). Furthermore, most of the participants reported being single (70.1%), having a middle school diploma (44.7%), being students (32.7%), and reported going to the gym and/or weightlifting as their main exercise activity (34.3%; see Table 1). Their recruitment was online through a call for participants posted on various social media platforms and utilizing snowball sampling [50], where an anonymous link took participants to a survey if they clicked on the link that was attached. The only inclusion criterion was that participants had to engage in exercise at least three times a week for a minimum of 30 min in each session, which is in line with the parameter used for the Italian validation of the Exercise Addiction Inventory [51]. The self-report measures together with a demographic questionnaire (i.e., gender, age, marital status, education, occupation) were administered online via the *Google Forms* platform after the participants had been informed about the general aim of the study and had given their informed consent (electronically). Ethics approval was obtained from the first author’s institutional Ethical Committee (approval number 005/2021).

### 2.2. Measures

#### 2.2.1. Family Adaptability and Cohesion Evaluation Scales-IV (FACES-IV)

The FACES IV [33,52] is a 42-item self-report measure that assesses family functioning based on the dimensions involved in the Circumplex Model of Marital and Family Systems [33]. Items are scored on a five-point Likert scale from 1 (*Strongly Disagree*) to 5 (*Strongly Agree*) and comprise six scales: two balanced (cohesion, e.g., “*Family members are involved in each other’s lives*”; flexibility, e.g., “*Our family tries new ways of dealing with problems*”) and four unbalanced (disengaged, e.g., “*We get along better with people outside our family than inside*”; enmeshed, e.g., “*We spend too much time together*”; rigid, e.g., “*There are strict consequences for breaking the rules in our family*”; chaotic, e.g., “*We never seem to get organized in our family*”). The Italian version [52] used in the present study showed acceptable reliability (cohesion, α = 0.86; flexibility, α = 0.74; enmeshed, α = 0.68; disengaged, α = 0.73; chaotic, α = 0.69; rigid α = 0.0.71). 

#### 2.2.2. Exercise Addiction Inventory (EAI) 

The EAI [6,51,53] is a six-item self-report measure used to assess the risk of exercise addiction. Items (e.g., “*If I have to miss an exercise session, I feel moody and irritable*”) are scored on a five-point Likert scale from 1 (*strongly* disagree) to 5 (*strongly agree*). The Italian version [51] used in the present study showed adequate reliability (α = 0.70).

#### 2.2.3. Body Image Concern Inventory (BICI)

The BICI [54,55] is a 19-item self-report measure used to assess dysmorphic body image concerns. Items are scored on a five-point Likert scale from 1 (*never*) to 5 (*always*) and provide both a total score and two subscale scores: dysmorphic symptoms (e.g., “*I am dissatisfied with some aspect of my appearance*”) and symptom interference (e.g., “*I have missed social activities because of my appearance*”). The Italian version [55] used in the present study showed excellent reliability (α = 0.94).

### 2.3. Data Analysis

SPSS (v. 21.0 for Windows) was used to analyse the data. A *p*-value below 0.05 value was set as a statistical significance threshold in the present study. The final dataset did not contain missing values because the online platform used did not allow the submission of surveys unless all items were answered. Descriptive statistics were performed. Pearson’s correlation analyses were used to assess the association between the variables. Then, a series of moderated moderated-mediation analyses, a regression-based approach, were carried out to explore the relationship between the dimensions of family functioning included in the FACES-IV (cohesion, flexibility, enmeshed, disengaged, chaotic, rigid) and exercise addiction, with the mediation of body image concerns and the moderated moderation of age and gender. The macro-program PROCESS [56] was used to test the moderated moderated-mediations by applying Model 18. Finally, the conditional effect of the models was tested through simple slopes by investigating the moderated relationship at the three different levels (−1SD, Mean, +1SD) of the moderators.

## 3. Results

Descriptive statistics for the sample and the variables are shown in Table 1 and Table 2, respectively. 

As reported in Table 2, the correlational analysis demonstrated significant and positive associations of exercise addiction with scores on the FACES-IV subscales: disengaged (*r* = 0.12, *p* < 0.05), enmeshed (*r* = 0.13, *p* < 0.05), and rigid (*r* = 0.16, *p* < 0.01), as well as with body image concerns (BICI; *r* = 0.32, *p* < 0.01). In turn, body image concerns were significantly and positively related with all the unbalanced FACES-IV subscales: disengaged (*r* = 0.16, *p* < 0.01), enmeshed (*r* = 0.26, *p* < 0.01), rigid (*r* = 0.15, *p* < 0.01), and chaotic (*r* = 0.29, *p* < 0.01). 

**Figure 1:**
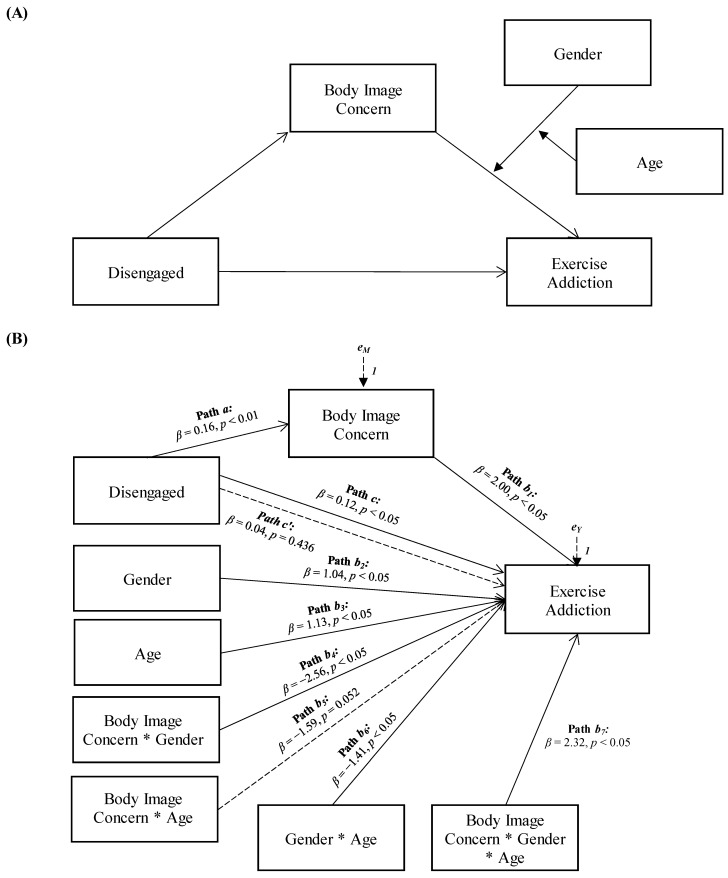
Statistical (**A**) and conceptual (**B**) forms of Model 1: A moderated moderated-mediation model involving disengaged family functioning, body image concern, gender, age, and exercise addiction.

**Figure 2:**
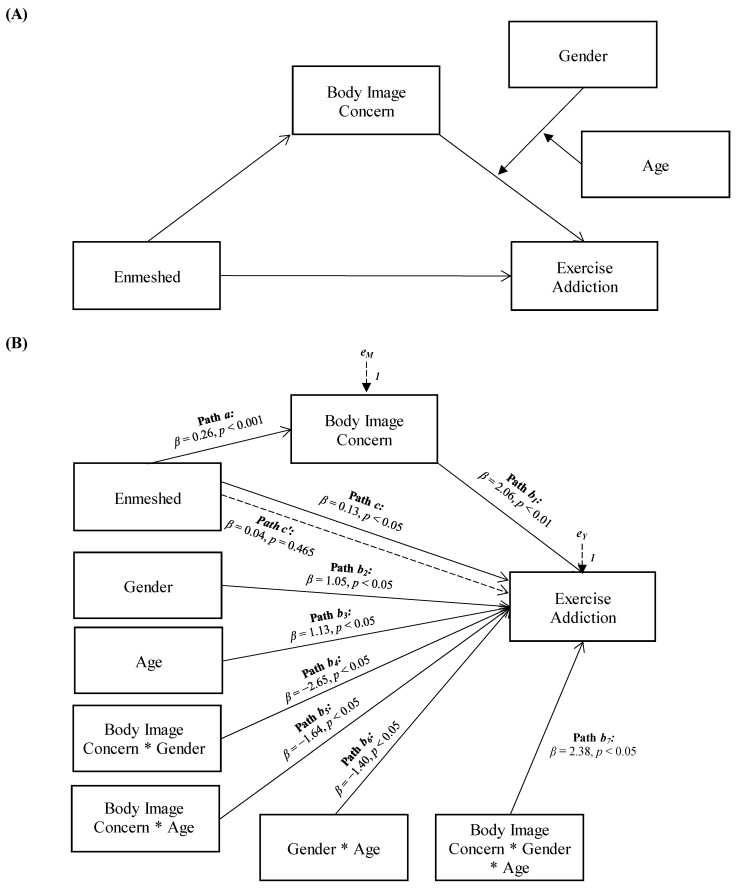
Statistical (**A**) and conceptual (**B**) forms of Model 2: A moderated moderated-mediation model involving enmeshed family functioning, body image concern, gender, age, and exercise addiction.

**Figure 3:**
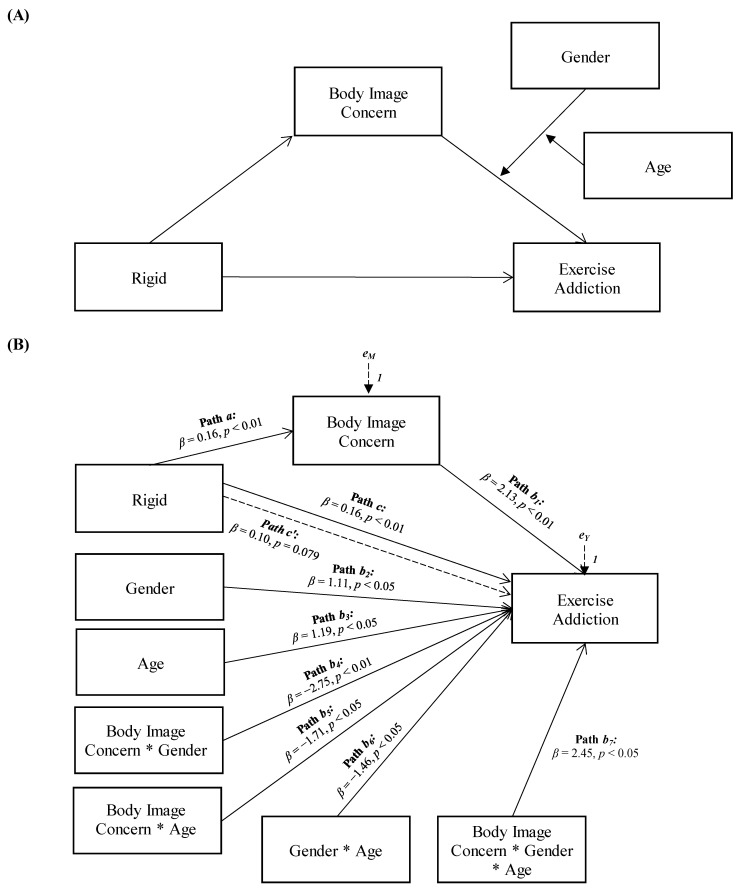
Statistical (**A**) and conceptual (**B**) forms of Model 3: A moderated moderated-mediation model involving rigid family functioning, body image concern, gender, age, and exercise addiction.

Concerning the moderated moderated-mediation analyses, no statistically significant models were identified for cohesion, flexibility, and chaotic family functioning since their total effects on exercise addiction were not significant (*β* = −0.04, *p* = 0.494; *β* = 0.65, *p* = 0.836; *β* = 0.04, *p* = 0.515, respectively). On the other hand, the models involving disengaged, enmeshed, and rigid family functioning patterns showed significant results (see Figure 1, Figure 2 and Figure 3). Specifically, body image concerns mediated the relationship between disengaged family functioning and exercise addiction, and the association between body image concerns and exercise addiction was moderated by the interaction between gender and age (see Figure 1).

More specifically, disengaged family functioning showed a significant and positive total effect on exercise addiction (Path *c* in Figure 1B; *β* = 0.12, *p* < 0.05, LLCI = 0.0065–ULCI = 0.2025). It was also significantly and positively associated with body image concerns (the mediator variable, path *a* in Figure 1B; *β* = 0.16, *p* < 0.01, LLCI = 0.1621–ULCI = 0.9268), which, in turn, was significantly related to exercise addiction (path *b*_1_ in Figure 1B; *β* = 2.00, *p* < 0.05, LLCI = 0.1223–ULCI = 0.8964). Furthermore, the effect of the mediator variable in exercise addiction was found to be significantly influenced by the interaction of the moderator variables, gender, and age (path *b*_7_ in Figure 1B; *β* = 2.32, *p* < 0.05, LLCI = −0.0012–ULCI = 0.0137): *ΔR*^2^ = 0.016, *F*(1, 291) = 5.478, *p* < 0.05. When included in the model, the moderated effect of body image concerns mediated the effect of disengaged family functioning on exercise addiction, reducing the direct effect, which becomes non-significant (path *c’* in Figure 1B; *β* = 0.04, *p* = 0.436, LLCI = −0.0585–ULCI = 0.1354): *R*^2^ = 0.141, *F*(8, 291) = 5.967, *p* < 0.001.

**Figure 4:**
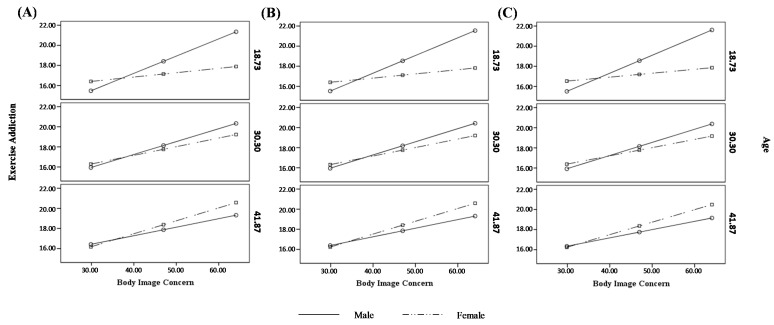
Graphic representation of the moderated-moderation effects for Model 1 (**A**), Model 2 (**B**), and Model 3 (**C**).

The moderation effect was further investigated by testing the conditional effects of the focal predictor for males and females at three levels of age (i.e., −1SD, mean, and +1SD). For male participants, the association between body image concerns and exercise addiction was slightly stronger at younger age (estimate = 0.17[0.05], *p* < 0.001, LLCI = 0.0723–ULCI = 0.2709) than at average age (estimate = 0.13[0.03], *p* < 0.001, LLCI = 0.0604–ULCI = 0.1960), and it became non-significant at an older age (estimate = 0.09[0.04], *p* = 0.051, LLCI = −0.0002–ULCI = 0.1699). For female participants, the association between body image concerns and exercise addiction was non-significant at younger ages (estimate = 0.04[0.02], *p* = 0.062, LLCI = −0.0022–ULCI = 0.0890), became significant at average age (estimate = 0.09[0.02], *p* < 0.001, LLCI = 0.0516–ULCI = 0.1204), and was stronger at an older age (estimate = 0.13[0.03], *p* < 0.001, LLCI = 0.0746–ULCI = 0.1827). Therefore, the positive indirect effect of disengaged family functioning on exercise addiction via body image concerns weakened for male and older participants, while increased for females with increasing age (see Figure 4A). 

Results also confirmed that body image concerns mediated the relationship between enmeshed family functioning and exercise addiction, and the association between body image concerns and exercise addiction was moderated by the interaction between gender and age (see Figure 2). 

More specifically, enmeshed family functioning showed a significant and positive total effect on exercise addiction (Path *c* in Figure 2B; *β* = 0.13, *p* < 0.05, LLCI = 0.0113–ULCI = 0.2272). It was also significantly and positively associated with body image concerns, the mediator variable (Path *a* in Figure 2B; *β* = 0.26, *p* < 0.001, LLCI = 0.5749–ULCI = 1.3979), which, in turn, was significantly related to exercise addiction (path *b*_1_ in Figure 2B; *β* = 2.06, *p* < 0.01, LLCI = 0.1377–ULCI = 0.9145). Furthermore, the effect of the mediator variable in exercise addiction was found to be significantly influenced by the interaction of the moderator variables, gender, and age (path *b*_7_ in Figure 2B; *β* = 2.38, *p* < 0.05, LLCI = −0.0013–ULCI = 0.0139): *ΔR*^2^ *=* 0.017, *F*(1, 291) = 5.685, *p* < 0.05). When included in the model, the moderated effect of body image concerns mediated the effect of enmeshed family functioning on exercise addiction, reducing the direct effect, which became non-significant (path *c’* in Figure 2B; *β* = 0.04, *p* = 0.465, LLCI = −0.0675–ULCI = 0.1475): *R*^2^ *=* 0.141, *F*(8, 291) = 5.957, *p* < 0.001. 

The moderation effect was further investigated by testing the conditional effects of the focal predictor for males and females at three levels of age (i.e., −1SD, mean, and +1SD). For male participants, the association between body image concerns and exercise addiction was slightly stronger at younger age (estimate = 0.18[0.05], *p* < 0.001, LLCI = 0.0769–ULCI = 0.2750) than at average age (estimate = 0.13[0.03], *p* < 0.001, LLCI = 0.0635–ULCI = 0.1980) and older ages (estimate = 0.09[0.04], *p* < 0.05, LLCI = 0.0007–ULCI = 0.1705). For female participants, the association between body image concerns and exercise addiction was non-significant at younger age (estimate = 0.04[0.02], *p* = 0.077, LLCI = −0.0046–ULCI = 0.0879), became significant at average age (estimate = 0.09[0.02], *p* < 0.001, LLCI = 0.0492–ULCI = 0.1199), and was stronger at an older age (estimate = 0.13[0.03], *p* < 0.001, LLCI = 0.0728–ULCI = 0.1822). Therefore, the positive indirect effect of enmeshed family functioning on exercise addiction via body image concerns weakened for male and older participants, while it increased for females with increasing age (see Figure 4B). Data showed that body image concerns also mediated the relationship between rigid family functioning and exercise addiction, and the association between body image concerns and exercise addiction was moderated by the interaction between gender and age (see Figure 3).

More specifically, rigid family functioning showed a significant and positive total effect on exercise addiction (Path *c* in Figure 3B; *β* = 0.16, *p* < 0.01, LLCI = 0.0378–ULCI = 0.2354). It was also significantly and positively associated with body image concerns (the mediator variable, path *a* in Figure 3B; *β* = 0.16, *p* < 0.01, LLCI = 0.1692–ULCI = 0.9434), which, in turn, was significantly related to exercise addiction (path *b_1_* in Figure 3B; *β* = 2.13, *p* < 0.01, LLCI = 0.1564–ULCI = 0.9297). Furthermore, the effect of the mediator variable on exercise addiction was found to be significantly influenced by the interaction of the moderator variables, gender, and age (path *b*_7_ in Figure 4B; *β* = 2.45, *p* < 0.05, LLCI = −0.0016–ULCI = 0.0141): *ΔR*^2^ *=* 0.018, *F*(1, 291) = 6.088, *p* < 0.05. When included in the model, the moderated effect of body image concerns mediated the effect of rigid family functioning on exercise addiction, reducing the direct effect, which became non-significant (path *c’* in Figure 3B; *β* = 0.10, *p* = 0.079, LLCI = −0.0100–ULCI = 0.1835): *R*^2^ *=* 0.148, *F*(8, 291) = 6.331, *p* < 0.001.

The moderation effect was further investigated by testing the conditional effects of the focal predictor for males and females at three levels of age (i.e., −1SD, mean, and +1SD). For male participants, the association between body image concerns and exercise addiction was slightly stronger at a younger age (estimate = 0.18[0.05], *p* < 0.001, LLCI = 0.0798–ULCI = 0.2772) than at average age (estimate = 0.13[0.03], *p* < 0.001, LLCI = 0.0634–ULCI = 0.1972), and it became non-significant at an older age (estimate = 0.08[0.04], *p* = 0.056, LLCI = −0.0022–ULCI = 0.1664). For female participants, the association between body image concerns and exercise addiction was non-significant at a younger age (estimate = 0.04[0.02], *p* = 0.0944, LLCI = −0.0067–ULCI = 0.0847), became significant at average age (estimate = 0.08[0.02], *p* < 0.001, LLCI = 0.0470–ULCI = 0.1162), and was stronger at an older age (estimate = 0.13[0.03], *p* < 0.001, LLCI = 0.0702–ULCI = 0.1781). Therefore, the positive indirect effect of rigid family functioning on exercise addiction via body image concerns weakened for male and older participants, while it increased for females with increasing age (see Figure 4B). The main indices of the models are summarized in Table 3. 

## 4. Discussion

Although physical exercise is recognized as an important factor for subjective well-being, it may become harmful when it becomes uncontrolled and acquires the characteristics of addiction [7,9,57,58]. Since pathological exercise compromises the psychophysical integrity of those affected [20,21], the study of the factors that can influence it is particularly important. Therefore, the present study explored the role of variables having an impact on exercise addiction, deepening the associations with the patterns of family functioning, body image concerns, age, and gender.

Among the dimensions of family functioning described in the Olson circumflex model [33], three out of four unbalanced patterns showed a significant and positive influence on exercise addiction. More specifically, chaotic family functioning did not exhibit a significant association with exercise addiction, aligning with findings observed in other behavioural addictions [34]. On the other hand, as the perception of family functioning as enmeshed, disengaged, or rigid increases, higher levels of exercise addiction were found. These findings appear consistent and enhance the pre-existing scientific literature on the role of family context in behavioural addictions [59], and are in line with previous research that has associated these patterns with problematic smartphone use [29], cyberpornography addiction [60], and problematic internet use [30]. Furthermore, these results further support the applicability of Olson’s circumflex model [33] in the research field of addiction. Indeed, the use of this model elucidates the role of the family context concerning this phenomenon by offering detailed profiles of family functioning. 

Moreover, the relationships between enmeshed, disengaged, and rigid patterns and exercise addiction were entirely mediated by body image concerns. This suggests that unbalanced family functioning mainly exerts its effect on exercise addiction indirectly. This further confirms the role of the family context in the development of body image [61] and the relevance of individuals’ dissatisfaction with their bodies in the association with exercise addiction [16,43]. In the models developed in the present study, these relationships were further detailed, as the effect of body image concerns on exercise addiction did not appear linear but rather was moderated by the interaction between age and gender. This finding could offer a reasonable perspective for understanding the apparent inconsistency between the results of previous studies regarding gender or age differences in exercise addiction [15,45,48], because, to the authors’ knowledge, there are no previous studies that have analysed these variables considering their mutual influence and in the relationship with body image concerns. 

More specifically, for females, the effect of body image concerns on exercise addiction was not significant for younger females (therefore making the entire indirect effect non-significant and neutralizing the influence of enmeshed, disengaged, and rigid family functioning on problematic exercise), while this positive relationship becomes increasingly strong with increasing age. For males, on the contrary, the effect of body image concerns on exercise addiction decreases with increasing age (becoming non-significant in the case of models regarding disengaged, and rigid family functioning, neutralizing their influence on problematic exercise). A possible explanation is that among females, a more satisfactory body image might be linked more to the aspects of thinness and weight control [44] which among younger individuals could mainly express themselves with other restrictive or compensatory behaviours, which is consistent with the higher likelihood of developing eating disorders among lower age groups found in the literature [62]. On the other hand, the greatest effects of body image concerns on exercise addiction among younger males may be due to their higher levels of impulsivity characterizing this life stage [63,64], which is one of the key elements in addictions [65] and shows a decreasing trend with increasing age [66,67]. 

The present study also has some limitations that should be noted. First, the cross-sectional design of the study implies the need to be cautious in interpreting causality. In future investigations, longitudinal studies should be carried out in order to validate the relationship between the analysed variables. Furthermore, the nature of the evaluation tools should be considered. Only self-report measures were used to collect the data, exposing well-known method biases, such as social desirability. Furthermore, the extant literature on the topic uses many different psychometric instruments to assess exercise addiction and almost all of the published studies have employed convenience sampling, and these can be a source of inconsistent findings. The application of a multimethod approach (e.g., by integrating self-report measures with interviews) could be a strategy for future research to overcome this issue. Moreover, the Cronbach’s alpha of the enmeshed subscale (α = 0.68) and chaotic subscale (α = 0.69) were below 0.70. Although previous evidence supports that an alpha value of 0.60 may be considered acceptable [68], future research should replicate our findings by using measures with higher internal consistency. In addition, this study’s significance criterion was set at *p* < 0.05 without performing correction procedures. Although there is a debate in the scientific literature in this regard, some authors support that *p*-value correction techniques may increase the risk of type II errors [69]. Therefore, these findings require confirmation in future research by more in-depth analyses. Moreover, the sample size was relatively modest and over two-thirds of the sample were female. 

Also, the data were collected on the internet via snowball sampling. Therefore, the sample may not be representative of the entire population of regular exercisers (e.g., those who do not have internet access may be unrepresented in the present study). An important challenge for future research should be to integrate the findings here with studies using a more inclusive gender-balanced sample. Additionally, no detailed information on relationship status was collected (e.g., how long the participants had been single or in a relationship, whether or not they were satisfied with their relationship status, whether or not they were looking for a new relationship at the time of the study). Since previous research has shown that individuals not engaged in a romantic relationship might have stronger concerns about their appearance [70], the influence of this variable on exercise addiction should be carefully examined in future research. 

Likewise, exploring how the demographic variables not considered in this research might predict exercise addiction could be examined in future studies. In line with this, the motivations associated with exercise and its dysregulated practice can be diverse (i.e., appearance, emotion regulation, etc.). Therefore, the exploration of other factors associated with this behavioural addiction is not confined to this research and should be expanded to include other elements considered in the field of addiction (e.g., attachment patterns) [71,72]. Finally, different studies use different criteria to define regular exercisers, and the effect of the amount of exercise or the type of sport might have on exercise addiction was not investigated in the present study. This may be an important element to consider, since research suggests that exercise addiction and its subsequent comorbidities are more likely to occur among individuals who exceed the World Health Organization’s guidelines on physical activity [73,74]. Future research needs to examine exercise frequency and history of exercise in relation to exercise addiction.

## 5. Conclusions

Behaviours such as work, sports, and shopping are socially legitimate and often recommended and encouraged. This makes it difficult for both patients and therapists to recognize the pathological elements of these activities [75]. Behavioural addictions represent a serious problem for the health of those affected and therefore it is important to study in-depth the risk/protective factors and to prepare specific intervention and/or prevention programs. The present study focused on specific variables associated with exercise addiction, providing insight into the role of family functioning patterns, body image concerns, age, and gender. 

The obtained findings may provide useful practical implications. Indeed, adopting Olson et al.’s model [33] provides detailed information on dysfunctional family functioning patterns which may play a greater role as risk factors for exercise addiction. Complementarily, this perspective can be a guide to orient tailored therapeutic interventions from a systemic perspective. Additionally, the results support the utility of direct therapeutic work towards the improvement of body image perception. Finally, the analysis of the role of gender and age can provide valuable insights into the population groups most at risk, guiding preventive programs. In conclusion, findings from the present study may provide insight to stimulating further research in the field, as well as for specialists from the sports training field to protect the well-being and psychophysical health of regular exercisers.

## Figures and Tables

**Table 1:** Demographic characteristics of the sample (*N* = 300).

Characteristics		*M* ± *SD*	*n*	%
*Age* (years)		30.3 ± 11.57		
*Gender*				
	Females		209	69.7
	Males		91	30.3
*Marital Status*				
	Single		212	70.7
	Married		38	12.7
	Cohabiting		37	12.3
	Separated		7	2.3
	Divorced		5	1.7
	Widowed		1	0.3
*Education*				
	Middle school diploma		13	4.3
	High school diploma		134	44.7
	University degree		86	28.7
	Master’s degree		42	14.0
	Post-lauream specialization		25	8.3
*Occupation*				
	Student		98	32.7
	Working student		56	18.7
	Employee		84	28.0
	Freelance		18	6.0
	Entrepreneur		16	5.3
	Trader		6	2.0
	Artisan		3	1.0
	Armed forces		1	0.3
	Unemployed		11	3.7
	Retired		7	2.3
*Sport*				
	Climbing		5	1.7
	Cycling or motorcycling		7	2.3
	Dance		21	7.0
	Football/Soccer		20	6.7
	Gym and/or weightlifting		103	34.3
	Martial arts and combat sports		10	3.3
	Pole dancing, aerial silks workouts, and calisthenics		16	5.3
	Running or athletics		23	7.7
	Swimming		34	11.3
	Triathlon or more sports		12	4.0
	Volleyball, water polo, basketball, or rugby		28	9.3
	Walk and trekking		16	5.3
	Others		5	1.7

**Table 2:** Correlations and descriptive statistics of the variables.

	1	2	3	4	5	6	7	8
1. Exercise addiction	1							
2. Body image concern	0.32 **	1						
*(family functioning)*								
3. Cohesion	−0.04	−0.08	1					
4. Flexibility	0.01	−0.06	0.79 **	1				
5. Disengaged	0.12 *	0.16 **	−0.60 **	−0.45 **	1			
6. Enmeshed	0.13 *	0.26 **	0.04	0.10	0.17 **	1		
7. Rigid	0.16 **	0.16 **	0.07	0.29 **	0.20 **	0.50 **	1	
8. Chaotic	0.04	0.29 **	−0.22 **	−0.26 **	0.42 **	0.33 **	0.10	1
*M*	17.7	47.0	26.3	24.1	16.7	14.0	17.5	16.5
*SD*	4.3	17.1	6.0	5.2	5.0	4.6	5.0	5.0

***Note***: Bold values indicate significant *p*-values. ** Correlation is significant at the 0.01 level (2-tailed). * Correlation is significant at the 0.05 level (2-tailed).

**Table 3:** Main indices of the models.

Antecedent	Total Effect (B)	Direct Effect (B)	Indirect Effect (B)						Test of Highest-Order Unconditional Interaction:
			Male			Female			
			−1SD ^a^	Mean ^a^	+1SD ^a^	−1SD ^a^	Mean ^a^	+1SD ^a^	
Disengaged family functioning	0.11 *	0.04	0.09 *	0.07 *	0.05	0.02	0.05 *	0.07 *	*ΔR*^2^ = 0.016 * F(1, 291) = 5.478, *p* < 0.05
Enmeshed family functioning	0.12 *	0.04	0.17 *	0.13 *	0.08 *	0.04	0.08 *	0.14 *	*ΔR*^2^ = 0.017 * F(1, 291) = 5.685, *p* < 0.05
Rigid family functioning	0.14 *	0.09	0.10 *	0.07 *	0.05	0.02	0.05 *	0.07 *	*ΔR*^2^ = 0.018 * F(1, 291) = 6.088, *p* < 0.05

***Note:*** * The effect is significant; ^a^ level of age.

## Data Availability

The data used in this study are available upon request from the corresponding author.
